# ITE and TCDD Differentially Regulate the Vascular Remodeling of Rat Placenta via the Activation of AhR

**DOI:** 10.1371/journal.pone.0086549

**Published:** 2014-01-24

**Authors:** Yanming Wu, Xiao Chen, Qian Zhou, Qizhi He, Jiuhong Kang, Jing Zheng, Kai Wang, Tao Duan

**Affiliations:** 1 Clinical and Translational Research Center, Shanghai First Maternity and Infant Hospital, Tongji University School of Medicine, Shanghai, P.R. China; 2 Department of Obstetrics, Shanghai First Maternity and Infant Hospital, Tongji University School of Medicine, Shanghai, P.R. China; 3 Department of Pathology, Shanghai First Maternity and Infant Hospital, Tongji University School of Medicine, Shanghai, P.R. China; 4 Shanghai Key Laboratory of Signaling and Disease Research, School of Life Science and Technology, Tongji University, Shanghai, P.R. China; 5 Department of Ob/Gyn, University of Wisconsin, Madison, Wisconsin, United States of America; Harvard Medical School, United States of America

## Abstract

Vascular remodeling in the placenta is essential for normal fetal development. The previous studies have demonstrated that in utero exposure to 2,3,7,8-tetrachlorodibenzo-*p*-dioxin (TCDD, an environmental toxicant) induces the intrauterine fetal death in many species via the activation of aryl hydrocarbon receptor (AhR). In the current study, we compared the effects of 2-(1′H-indole-3′-carbonyl)-thiazole-4-carboxylic acid methyl ester (ITE) and TCDD on the vascular remodeling of rat placentas. Pregnant rats on gestational day (GD) 15 were randomly assigned into 5 groups, and were exposed to a single dose of 1.6 and 8.0 mg/kg body weight (bw) ITE, 1.6 and 8.0 µg/kg bw TCDD, or an equivalent volume of the vehicle, respectively. The dams were sacrificed on GD20 and the placental tissues were gathered. The intrauterine fetal death was observed only in 8.0 µg/kg bw TCDD-exposed group and no significant difference was seen in either the placental weight or the fetal weight among all these groups. The immunohistochemical and histological analyses revealed that as compared with the vehicle-control, TCDD, but not ITE, suppressed the placental vascular remodeling, including reduced the ratio of the placental labyrinth zone to the basal zone thickness (at least 0.71 fold of control), inhibited the maternal sinusoids dilation and thickened the trophoblastic septa. However, no marked difference was observed in the density of fetal capillaries in the labyrinth zone among these groups, although significant differences were detected in the expression of angiogenic growth factors between ITE and TCDD-exposed groups, especially Angiopoietin-2 (Ang-2), Endoglin, Interferon-γ (IFN-γ) and placenta growth factor (PIGF). These results suggest ITE and TCDD differentially regulate the vascular remodeling of rat placentas, as well as the expression of angiogenic factors and their receptors, which in turn may alter the blood flow in the late gestation and partially resulted in intrauterine fetal death.

## Introduction

Normal placental vascular growth and remodeling are required for transferring oxygen, nutrients and metabolites between maternal system and fetal system to support fetal growth [1]. Any impaired placental vascular development will lead to adverse effects on both mother and fetus, possibly leading to preeclampsia, abortion and intrauterine growth retardation [1]–[4]. In rats, the rapid placental vascular growth and remodeling occur between gestational day (GD)15 to GD20 as the placenta blood flow increases exponentially in order to nourish the rapid growth of fetus [5], [6]. Thus, it may provide useful information to study the placental vascular development in such an important phase by comparing the effects of environmental factors.

It is well documented that placental vasculature develops through two consecutive processes: vasculogenesis and angiogenesis [7]–[9]. During the vasculogenesis, the endothelial progenitor cells are assembled to form a primitive vascular network. The term angiogenesis represents the process of sprouting and remodeling of new vessels from the pre-existing vascular network into a complex vascular network [8], [9]. The establishment and remodeling of the placental blood vessels are regulated by a broad spectrum of angiogenic-associated factors and their receptors, including of vascular endothelial growth factor (VEGF)/VEGF receptor (VEGFR) system and Angiopoietin (Ang)/Tie2 system and cytokines, etc. In addition, placental vascular development can be stimulated under a hypoxic condition because the activation of HIF-1α not only leads to the adaptation of hypoxia, but also activates the transcription of VEGF/VEGFR system and regulates the expression of VEGF and fms-like tyrosine kinase-1 (Flt-1) [7], [10], [11].

The aryl hydrocarbon receptor (AhR) is a ligand-inducible transcriptional regulator which is highly expressed in human placentas and many fetal organs during fetal development [12], [13]. Upon binding to its ligands, the AhR translocate into the nucleus and binds to its heterodimer aryl hydrocarbon receptor nuclear translocator (ARNT), inducing the expression of downstream genes, such as CYP1A1 and CYP1B1 (encoding cytochromes P450 1A1 and 1B1). It was reported that activated CYP1 enzymes are responsible for both metabolizing and detoxifying numerous environmental contaminants collectively called aryl hydrocarbons (AHs) [14], [15]. 2,3,7,8-tetrachlorodibenzo-*p*-dioxin (TCDD) is the most potent toxicant among dioxins and related environmental pollutants. It is released unintentionally in industrial processes, waste combustion, metal smelting, etc. Because of its resistance to metabolic breakdown and accumulation in biologic chain, TCDD can cause many toxic effects on human and animal health, including teratogenicity, immunotoxicity and various metabolic dysfunctions [14], [16], [17]. Among all the adverse events of TCDD, intrauterine fetal death is the most common in mammals as the fetus is much more sensitive to TCDD when compared with the mother [7], [18], [19]. Convincing evidence from laboratory animal studies has shown that TCDD affects placental angiogenesis and vascular remodeling during embryo and fetal development by reducing blood flow and circulatory function [7], [19]–[21]. Considering the highly conserved structure of AhR, many endogenous AhR ligands have been discovered, including 2-(1′H-indole-3′-carbonyl)-thiazole-4-carboxylic acid methyl ester (ITE), which was first isolated from porcine lungs [22]. From preliminary studies, ITE exhibits its high affinity to AhR both *in vivo* and *in vitro* as well as TCDD [16], [23], moreover, it has been reported that nontoxic ITE suppresses the development of experimental autoimmune encephalomyelitis (EAE) by promoting the induction of functional Treg cells *in vivo* [24]. However, ITE does not cause adverse effects on fetus like TCDD does [16].

Considering the toxic outcomes of TCDD on both pregnant women and fetus, in the present study, we try to use ITE as a nontoxic, endogenous control to imitate the physiological activation of AhR and compare the potential differences between ITE and TCDD on the vascular remodeling and expression of angiogenic-associated factors in the rat placentas. The results suggest that ITE and TCDD differentially regulate the placental vascular remodeling, as well as the profile of angiogenic factors and their receptors through the activation of AhR.

## Materials and Methods

### Reagents

TCDD (purity: >99.2%) was purchased from Cambridge Isotope Laboratory (Andover, MA, USA). ITE (purity: >98%) was purchased from Tocris Bioscience (San Diego, CA, USA). Corn oil was obtained from Sigma-Aldrich (St. Louis, MO, USA). Trizol was obtained from Life Technologies (Rockville, MD, USA).

### Animals

Male and female Sprague-Dawley rats were purchased from Slac laboratory animal Inc. (Shanghai, China). The animals were maintained in a controlled environment with a 12 h light/12 h dark cycle and given free access to a solid diet and distilled water. Female rats were mated when they were 8–10 weeks. Female rats in proestrus were allowed to mate 2∶1 with males overnight. GD0 was designated after the vaginal plugs were observed next morning. All rats were randomly assigned to each group. ITE and TCDD were dissolved in DMSO (Sigma-Aldrich) at a stock concentration of 10 mM and 10 µM, respectively, and further diluted with corn oil. The pregnant rats were exposed to ITE (1.6 and 8.0 mg/kg bw), TCDD (1.6 and 8.0 µg/kg bw) or an equivalent of corn oil (vehicle-control) on GD 15 by oral gavage [16]. The control group, ITE-exposed groups and TCDD-exposed groups consisted of at least six pregnant rats. Dams were euthanized for the tissue collection on GD20 between 12∶00 p.m. and 1∶00 p.m. The placentas were collected. A portion of placental tissues were fixed in formaldehyde solution and embedded in paraffin for immunohistochemistry as described [12]. Additional placentas were immediately frozen in liquid nitrogen and maintained at −80°C until analysis.

### Histology and Immunohistochemistry

The middle part of the placenta was cut vertically and embedded in paraffin. The tissue sections were at approximately 5 µm. These sections were subjected to hematoxylin and eosin (HE) staining for morphological analysis. Additional sections were immunostained using CD31 (R&D Systems, MN, USA) and DAPI (Beyotime, Beijing, China) antibody. The CD31 was used as an endothelial marker for quantifying fetal capillaries. Five placentas each from different groups were randomly selected from five dams. The thickness analysis of five placentas of each group is exhibited as mean ± S.E.M.

### RNA Isolation and Real-time PCR

Total RNA of placenta from each group was extracted using Trizol and RNA simple Total RNA Kit (Tiangen Biotech, Beijing, China). RNA was quantified by UV absorption measure and 1 µg was reverse transcribed to cDNA. The reverse transcription (RT) was carried out using PrimeScript RT reagent Kit (TaKaRa, Dalian, China) for 15 min at 37°C, 5 sec at 85°C in a 20 µl reaction volume. Real-time PCR was performed using SYBR Premix Ex Taq (TaKaRa) according to the manufacturer’s instruction. Primer sequences were ordered from Sangon (Shanghai, China) (Table 1). The real-time PCR cycling condition for GAPDH, CYP1A1, VEGF-A, VEGF-B, PIGF, Flt-1, fetal liver kinase-1 (Flk-1), soluble Flt-1 (sFlt-1), HIF-1α, Endoglin and IFN-γ is 30 sec at 95°C for incubation, 40 cycles of 15 sec at 95°C and 20 sec at 60°C. To confirm the amplification specificity, the PCR products were subjected to a melting curve analysis. Levels of mRNA expression were analyzed using 2^−△△CT^ method and gene expression was normalized to GAPDH.

**Table 1:** Real-time PCR primers.

Target genes	Forward primer	Reverse primer	Product size (bp)
GAPDH a	5′-TGAAGCAGGCATCTGAGGG-3′	5′-CGAAGGTGCAAGACTCGGAG-3′	618
CYP1A1	5′-CCATGACCAGGAACTATGGG-3′	5′-TCTGGTGAGCATCCAGGACA-3′	341
VEGF-A	5′-AGAAACCCAATGAAGTGGTG-3′	5′-ACTCCAGGGCTTCATCATTG-3′	177
VEGF-B	5′-TGGTACCTCTGAGCATGGAA-3′	5′-GAGGATCTGCATTCGGACTT-3′	149
Flt-1	5′-CAAGGGACTCTACACTTGTC-3′	5′-CCGAATAGCGAGCAGATTTC-3′	239
Flk-1	5′-GCCAATGAAGGGGAACTGAAGAC-3′	5′-TCTGACTGCTGGTGATGCTGTC-3′	538
sFlt-1	5′-ACGTCACAGATGTGCCAAAC-3′	5′-CAACACAGGACAGTTTCAGG-3′	84
Ang-1	5′-GCCACTTGAGAATTACATTGTGG-3′	5′-CGCGGATTTTATGCTCTAATCAACTG-3′	305
Ang-2	5′-GACCAGTGGGCATCGCTACG-3′	5′-CATTGTCCGAATCCTTTGTGCT-3′	203
Tie-2	5′-CGGCCAGGTACATAGGAGGAA-3′	5′-TCACATCTCCGAACAATCAGC-3′	67
HIF-1α	5′-AGTCGGACAGCCTCAC-3′	5′-TGCTGCCTTGTATGGGA-3′	436
PIGF	5′-CCCACCTGGATGCTGTT-3′	5′-ATAGAGGGTAGGTACCAGCA-3′	119
Endoglin	5′-ACTGCACTTGGTCTACAACTC-3′	5′-TGCTATGGAGGTAATGGTGC-3′	136
IFN-γ	5′-CATTCATGAGCATCGCCAAG -3′	5′- ATCAGCACCGACTCCTTTTC-3′	135

a Primer sets for real-time PCR analysis.

### Statistical Analysis

SigmaStat, version 3.5 (Jandel Co., San Rafael, CA) was used for the statistical analyses. All the data were represented as the means ± S.E.M. Data for comparison of the birth outcome on GD20 were analyzed by the χ^2^ test. For comparison of placental weight, fetal weight, ratio of the labyrinth zone to the basal zone on GD20 and mRNA expression among these groups, Dunnett’s multiple comparison post hoc tests was carried out. Differences were considered to be statistically significant when the *p* value was less than 0.05.

### Ethics Statement

All experiments for animals were approved by the Animal Ethics and Experimentation Committee of the Tongji University, Shanghai, China, and were performed in accordance with the Guide for the Care and Use of Laboratory Animals published by the National Institutes of Health (publication no. 85–23, revised 1996).

## Results

### Effects of ITE or TCDD on Rats Reproductive Outcomes

**Figure 1:**
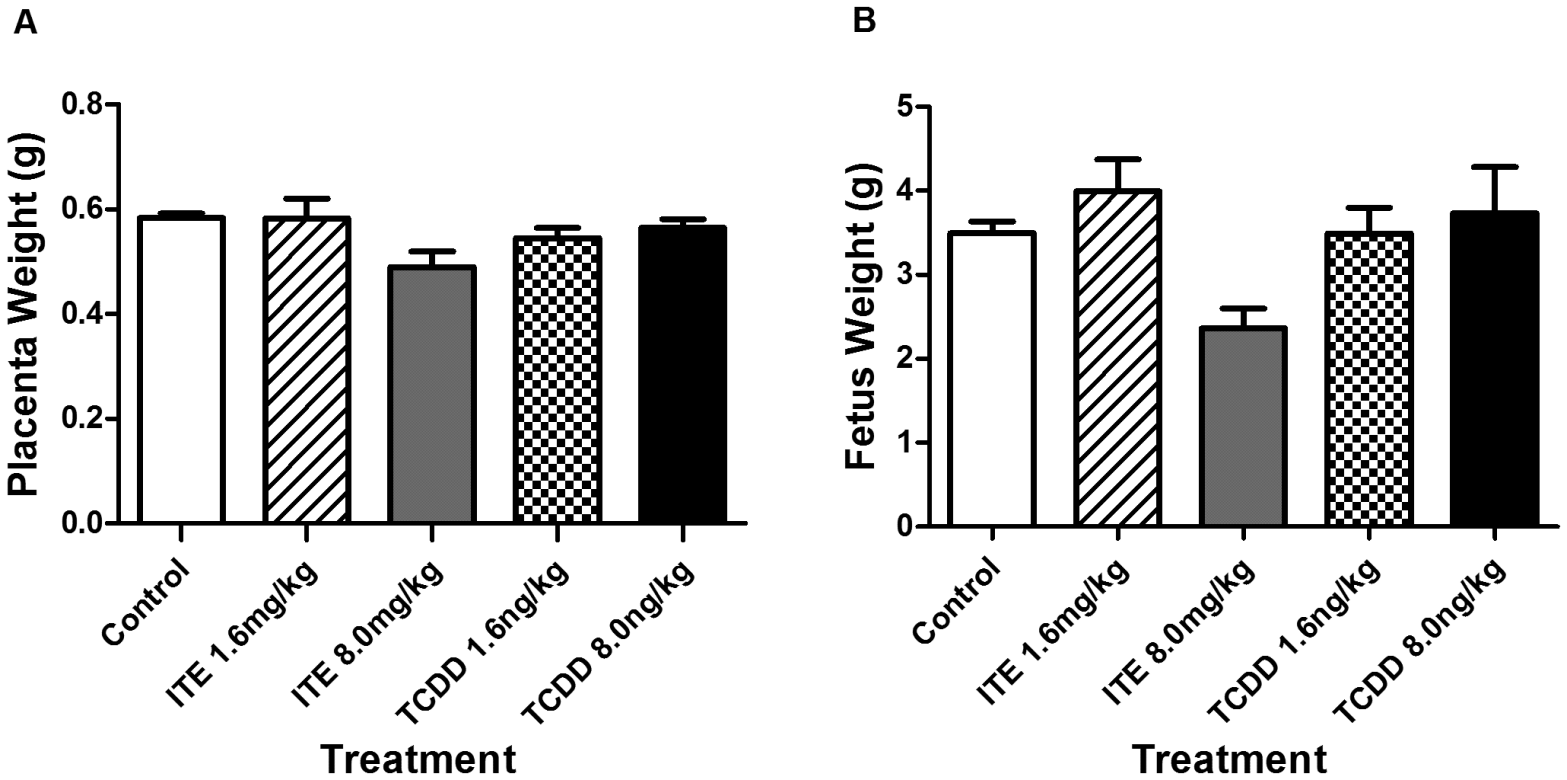
Effects of ITE and TCDD on placental (A) and fetual weights (B). The pregnant rats on GD15 were given ITE, TCDD or the vehicle-control (corn oil). Animals were euthanized on GD20. Placental and fetual weights were recorded. No significant difference was observed among the vehicle-control, ITE and TCDD treatment groups (*p*>0.05). Data are expressed as means ± S.E.M. (at least five placentas or fetus were counted in each group).

In the present study, we compared the differential reproductive outcomes after exposure of ITE or TCDD to Sprague-Dawley Rats. The dams’ death was only observed in 8.0 µg/kg bw TCDD-exposed group, in which the dams had pale eye color and underwent prone position on the morning of GD20 before parturition. There was no impairment and detectable abnormality in daily behavior, general condition, or food consumption in the dams treated with 8.0 µg/kg bw TCDD compared with other groups during pregnancy. No fetal death occurred by exposure to 1.6, 8.0 mg/kg bw ITE and 1.6 µg/kg bw TCDD. However, 21% of dead fetuses per litter (11/53, dead/born) were observed in 8.0 µg/kg bw TCDD-exposed group (Table 2). Both the fetus’ and dams’ death were evaluated based on the low body temperature and respiratory status. Moreover, the placental and fetal weights were similar among all ITE-exposed, TCDD-exposed groups and vehicle-control (Fig. 1 A–B). These indicate that ITE serves as a nontoxic agent which does not induce intrauterine fetal death even though the low dose of TCDD might not be enough to cause adverse effects on rat reproductive outcomes compared to the higher dose of TCDD.

**Table 2:** Effect of GD15 ITE/TCDD exposure on reproductive outcomes on GD20.

	DMSO	ITE (mg/kg)		TCDD (µg/kg)	
	(Vehicle)	1.6	8.0	1.6	8.0
Number of dams	8	8	6	6	9
Number of total fetuses	94	96	100	71	53
Number of fetuses per litter^a^	11.75±0.80	12.00±0.78	13.38±2.26	11.83±1.01	10.60±0.87
Total number of dead dam	0	0	0	0	4*
Total number of dead fetuses	0	0	0	0	11**
Number of dead fetuses per litter	0	0	0	0	2.2
Percent of dead fetuses per litter (%)	0	0	0	0	21

a Values represent means ± S.E.M. for the litters of six to nine dams.

The statistical significance of difference from vehicle-treated controls was evaluated by χ^2^ test (*p<0.05, **p<0.001).

### The Activation of AhR by Exposure to ITE or TCDD

**Figure 2:**
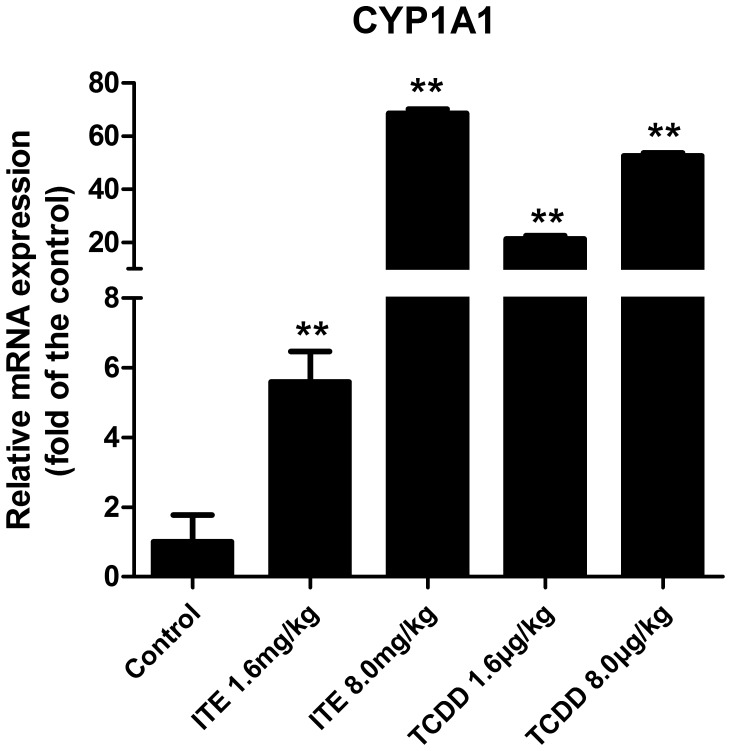
The activation of AhR/CYP1A1 signaling by ITE or TCDD in placental tissues. Activation of the AhR/CYP1A1 signaling was indicated by increases in CYP1A1 mRNA levels. Data are expressed as means ± S.E.M fold of the control. **Differ from the control (*p*<0.001; n = 4/group).

To examine the transcriptional activity of AhR in response to ITE or TCDD, we investigated the induction levels of CYP1A1 mRNA in the placentas collected on GD20 after exposed to administered TCDD and ITE on GD15 using real-time PCR. It was observed that ITE at 1.6 and 8.0 mg/kg bw, TCDD at 1.6 and 8.0 µg/kg bw significantly (*p*<0.05) increased the CYP1A1 mRNA levels by 5.59, 68.55, 21.26, and 52.52 fold, respectively, (Fig. 2), indicating the AhR activation of ITE and TCDD in placentas.

### Histological Examination of Placental Tissues

**Figure 3:**
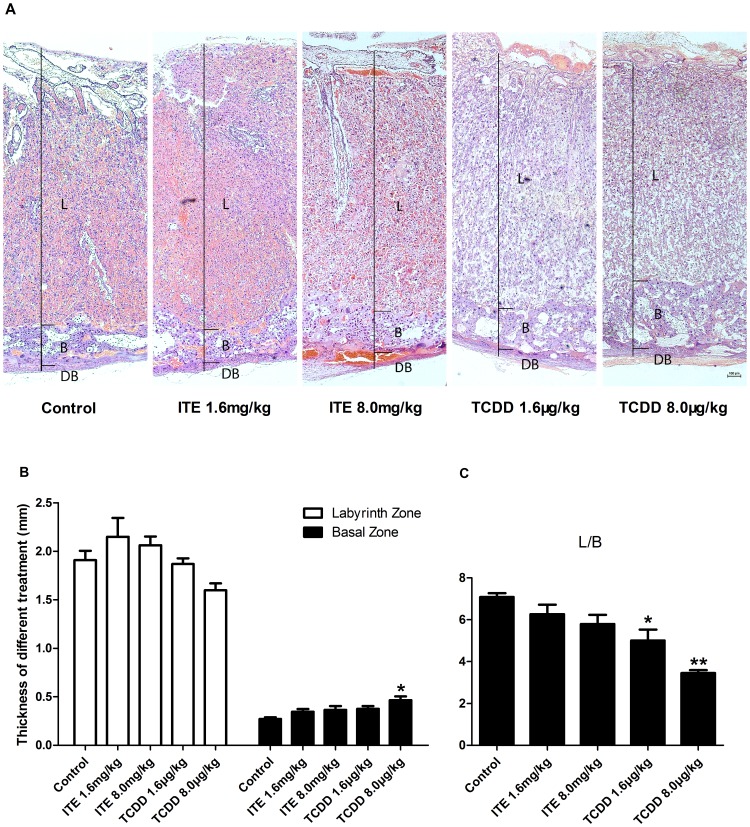
Effects of ITE and TCDD on placental morphology. A. HE staining for rat placentas (L, labyrinth zone; B, basal zone; DB, decidua basalis). Bar, 100 µm. B. Quantification of thickness of the labyrinth and the basal zone. C. Ratio of the labyrinth zone to the basal zone. *Differ from the control (*p*<0.050), **differ from the control (*p*<0.001; n = 5/group). Data are expressed as means ± S.E.M.

Several studies have shown that exposure of TCDD leads to the alteration on the rat placenta growth and interfere with the placental vascular remodeling [19]–[21]. However, little is known about the effects of ITE on rat placental development. Therefore, we examined if ITE altered placental morphologies as compared to TCDD. Our histological results showed that TCDD at 8.0 µg/kg bw (*p* = 0.009), but not ITE and TCDD at 1.6 µg/kg bw, initiated a delay of basal zone regression by increasing the thickness of the basal zone on GD20 as compared with the vehicle-control (Fig. 3A–B). Further analysis demonstrated that TCDD at 1.6 and 8.0 µg/kg bw decreased the ratio of the labyrinth zone to the basal zone on GD20 (*p = 0.026, **p<0.001) (Fig. 3C), although no significant difference was observed in the thickness of the labyrinth zone in all these four groups (*p* = 0.100) (Fig. 3B). These data suggest that in utero exposure to TCDD, instead of ITE, had a delay of placental development than those in the vehicle-control [25].

**Figure 4:**
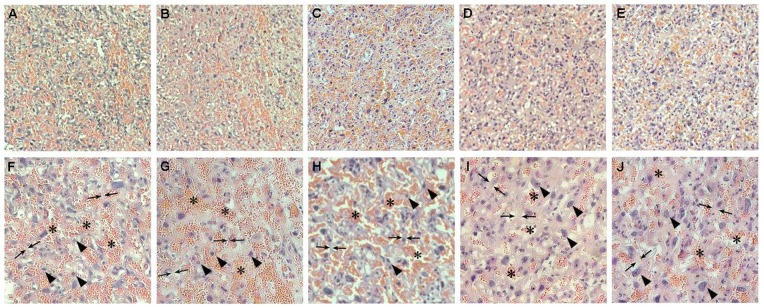
Effects of ITE and TCDD on distribution of RBCs and trophoblastic septa. The representative images of the placental labyrinth zone sections from control (A and F), 1.6 mg/kg bw ITE (B and G), 8.0 mg/kg bw ITE (C and H), 1.6 µg/kg TCDD (D and I) and 8.0 µg/kg TCDD group (E and J) are shown. The sections were subjected to HE staining. Asterisks indicate the maternal sinusoids that are occupied by RBCs, arrowheads indicate the trophoblasts and arrows indicate the trophoblastic septa. Magnification×100 (A, B, C, D, E) and×200 (F, G, H, I and J).

Considering that ITE and TCDD differentially altered fetal mortality in rats and retarded placental development, vertical sections of placentas from each group were prepared to analyze the effects of different dosage ITE and TCDD on the vasculature of the placental labyrinth zone. In the vehicle-control and both ITE-exposed group on GD20, the maternal sinusoids were similarly full of red blood cells (RBCs) (Figs. 4A–C; 4F–H, Asterisks). However, TCDD at both doses used appeared to constrict the maternal sinusoids, decrease the number of RBCs, (Figs. 4D–E; 4I–J, Asterisks), and increased the thickness of the trophoblastic septa (Figs. 4I–J, Arrows) when compared to either vehicle-control group or ITE-exposed groups (Figs. 4F–H, Arrows). These reduced maternal sinusoids were probably partially due to the enlargement of the placental trophoblasts (Figs. 4I–J, Arrowheads) and thickening of the trophoblastic septa of the placentas in TCDD-exposed groups. Thus, we prompt to propose that ITE does not significantly affect the placental vasculature since it is considered to be a nontoxic endogenous AhR ligand [22].

### Expression of CD31 on the Placental Labyrinth Zone

**Figure 5:**
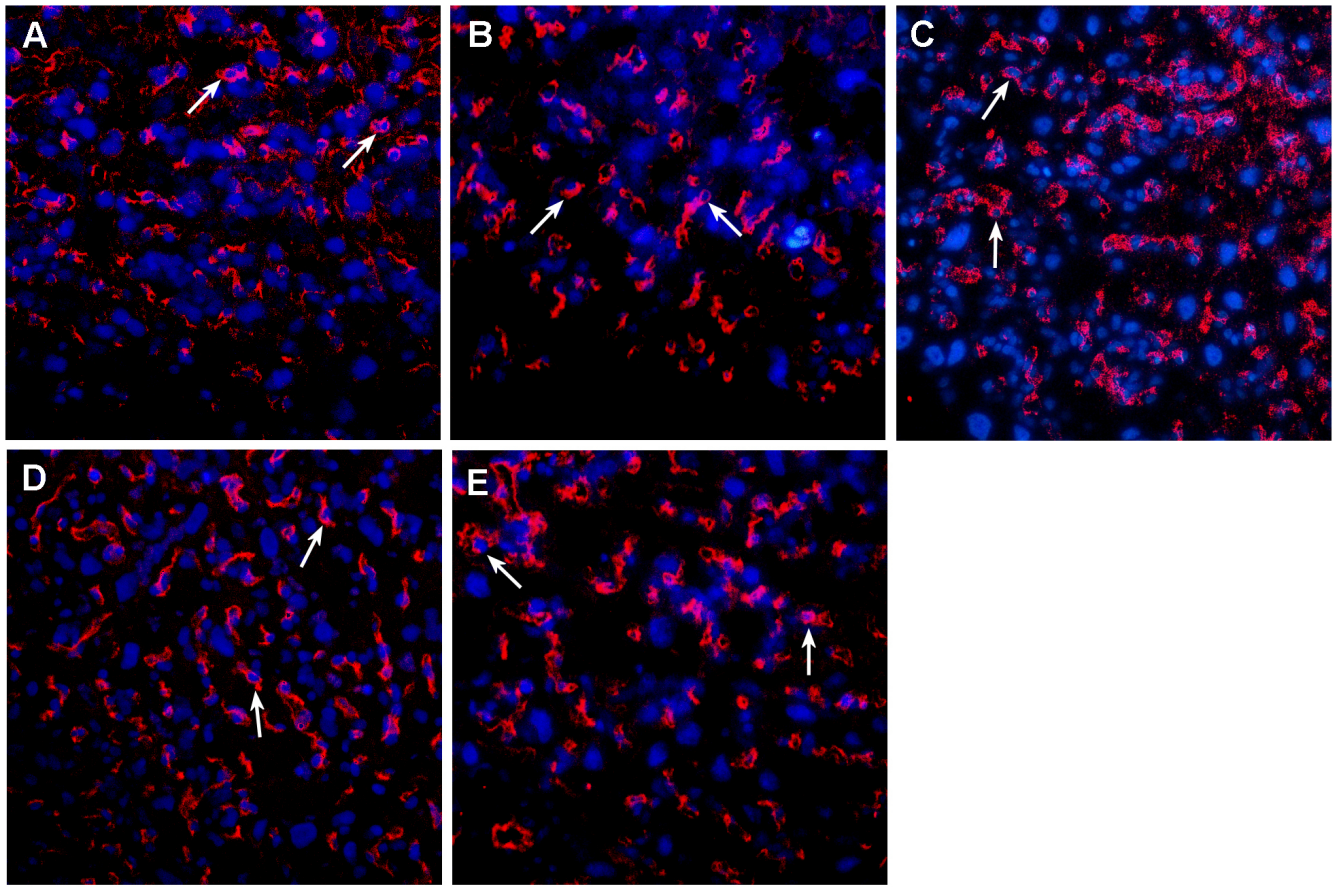
Expression of CD31 in the placental labyrinth zone from the control (A), 1.6 mg/kg bw ITE (B), 8.0 mg/kg bw ITE (C), 1.6 µg/kg TCDD group (D) and 8.0 µg/kg TCDD group (E). The tissue sections were probed with CD31 and countered with DAPI. Arrows indicate fetal capillaries in the trophoblastic septa. Magnification×200.

Since maternal sinusoids are full of maternal blood without an endothelium [26], we aimed to elucidate the effects of ITE or TCDD on fetal placental capillaries using CD31 as an endothelial marker [27]. Fig. 5 illustrated the expression of CD31 on the labyrinth zone on GD20. In digitized color images, the bright red color indicated positive CD31-labeled fetal capillaries and the blue DAPI color identified the nucleus. However, no significant difference was detected among the ITE, TCDD, and vehicle-control groups.

### Differential Expression of Angiogenic-associated Factors

**Figure 6:**
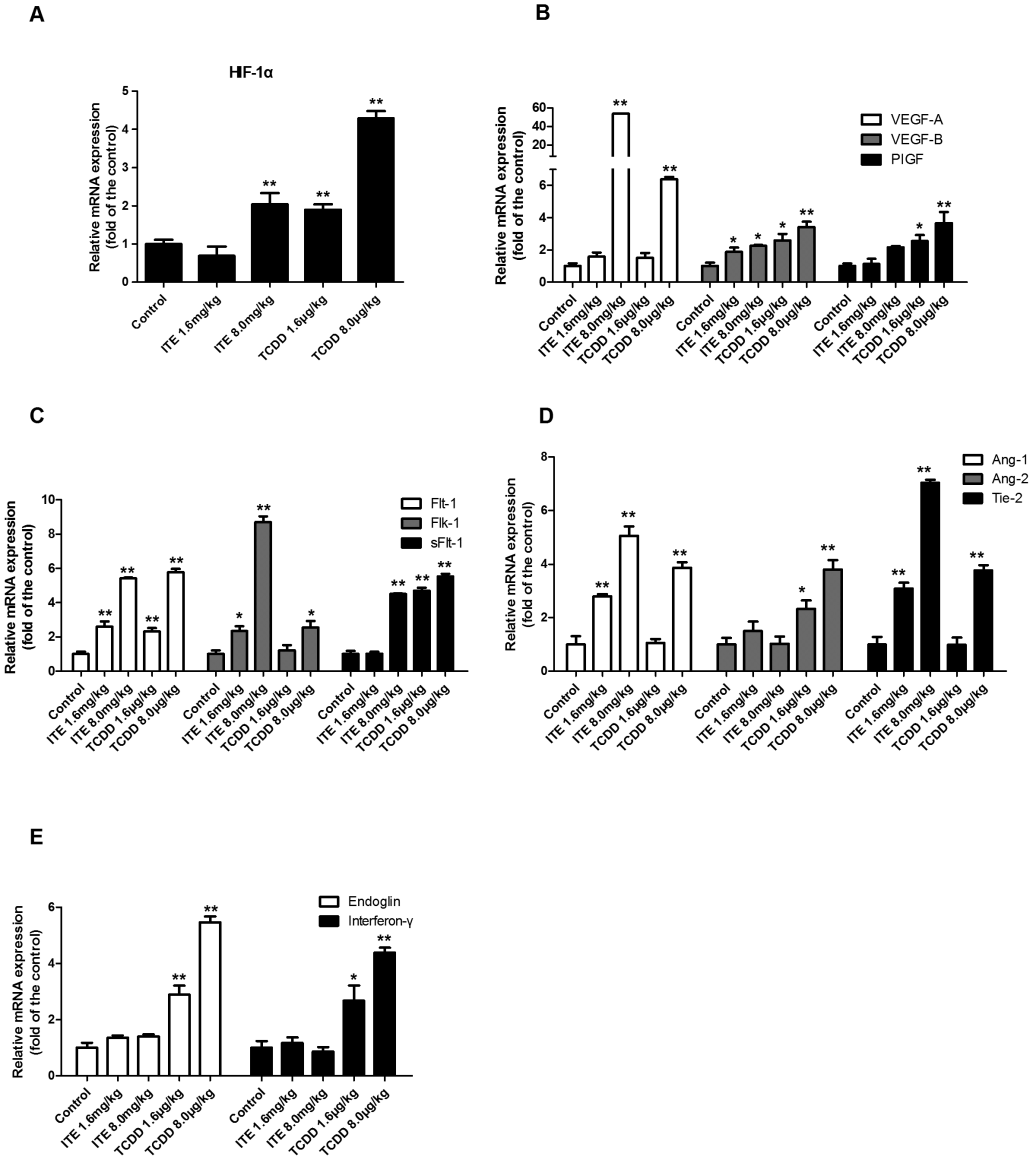
Effects of ITE and TCDD on mRNA expression of angiogenic-associated genes in placental tissues. The total RNA samples from placental tissues were subjected to real-time PCR. *Differ from the control (*p*<0.05), **differ from the control (*p*<0.001; n = 4/group). Data are expressed as means ± S.E.M fold of the control.

As ITE and TCDD differentially affect the placental vasculature on GD20 in rats, we hypothesized that the differential expression of angiogenic and anti-angiogenic factors, such as HIF-1α, VEGF/VEGFR system, Ang/Tie2 system, PIGF, Endoglin and IFN-γ contribute to these distinctions. The real-time PCR analysis revealed that the level of HIF-1α mRNA was elevated in 8.0 mg/kg bw ITE-exposed and both TCDD-exposed groups by 2.04, 1.90 and 4.29 fold (Fig. 6A). Regarding the VEGF/VEGFR system, the levels of VEGF-A, VEGF-B and PIGF mRNA were induced in a dose-dependent manner in ITE-exposed and TCDD-exposed groups, respectively. However, the inducibility of PIGF mRNA was only found in both 1.6 µg/kg and 8.0 µg/kg TCDD-exposed groups by 2.55 (*p* = 0.030) and 3.67 (*p*<0.001) fold and no significant difference in the expression of VEGF-A was found in 1.6 mg/kg bw ITE-exposed and 1.6 µg/kg bw TCDD-exposed group when compared with vehicle-control (Fig. 6B). The expression levels of Flt-1 and Flk-1 mRNA were significantly elevated in most ITE-exposed and TCDD-exposed group while the expression level of anti-angiogenic factor sFlt-1 mRNA was marked in 8.0 mg/kg bw ITE-exposed and both TCDD-exposed groups by at least 4.50 fold (*p*<0.001) (Fig. 6C). Regarding the Ang/Tie2 system, the expression levels of Ang-2 mRNA were significantly elevated in both 1.6 µg/kg and 8.0 µg/kg TCDD-exposed group by 2.34 (*p* = 0.018) and 3.80 (*p*<0.001) fold, whereas Ang-1 and Tie-2, the coreceptor of Ang-1 and Ang-2, were markedly elevated in both ITE-exposed and 8.0 µg/kg bw TCDD-exposed group (Fig. 6D). In addition, two anti-angiogenic factors, Endoglin and IFN-γ were also examined. Our data showed that the expression of Endoglin and IFN-γ mRNA were notably increased in TCDD-exposed groups by at least 2.89 and 2.68 fold, respectively, but not in ITE-exposed groups and vehicle-control (Fig. 6E). These indicate TCDD and ITE differentially induced the expression of angiogenic-associated factors which may partially attribute to the alteration of HIF-1α expression.

## Discussion

AhR is a ligand-activated transcription factor mediating the toxic effects of environmental pollutants such as TCDD. However, with a number of putative endogenous and natural AhR ligands (e.g., 6-formylindolo[3,2-b]carbazole (FICZ), ITE, and several arachidonic acid derivatives) have identified [22], [28], an increasing evidence emerged to show that AhR also has important physiological roles in reproduction, differentiation and immunity [12], although the mechanisms underlying these functions are as yet identified. In the current study, we present the first evidence showing that endogenous AhR ligand ITE and TCDD differentially regulated the rat placental vascular remodeling which was characterized by the dilation of maternal sinusoids and fetal capillaries [21], as well as expression of an array of angiogenesis related genes including angiogenic factors and their receptors.

It was reported that in utero exposure to TCDD resulted in fetal death at the late gestation in many animal species [7], [19]. Consistent with the previous studies, we observed the fetal death by exposure to 8.0 µg/kg bw TCDD with an incidence of 21%, whereas no adverse outcome was found in the other three groups. Thus, we provide convincing evidence that ITE has no toxic effect on placental and fetal survival, although the exposure to 1.6 µg/kg bw TCDD might not be enough to cause intrauterine fetal death regarding the lower dose or exposure duration.

It was demonstrated that the dose of ITE necessary to induce an approximately equivalent level of LacZ staining in the transgenic DRE-LacZ mouse fetuses and the transcription of AhR-responsive genes CYP1A1 and CYP1B1 were about 1000-fold greater than for TCDD [16], [23]. Considering the difference of uptake kinetics, binding and metabolism between ITE and TCDD, we administered 1.6 mg/kg bw ITE and 1.6 µg/kg bw TCDD, as well as a five-times higher dose of ITE and TCDD to examine its more obvious toxicities on the fetus and placental growth. Our observations showed that in utero exposure to ITE or TCDD significantly induced the transcription of CYP1A1, indicating the activation of AhR/CYP1A1 signaling in rat placentas [14], [16], [29]. The disparity of the induction level among ITE-exposed and TCDD-exposed groups might due to their different chemical stability and metabolism rate. This was consistent with the previous study that ITE is less potent than TCDD in inducing reporter gene activity both *in vivo* and *in vitro*, though ITE and TCDD have similar affinity and binding potency with AhR [16], [23].

In the present study, we observed that ITE and TCDD differentially regulated the development of the placental vascular remodeling, as indicated by the alteration of maternal sinusoids and trophoblastic septa in the labyrinth zone of the rat placenta. These results were consistent with recent study that TCDD significantly suppressed the dilation of maternal blood sinusoids in rats [21]. It has been well documented that the exchange of maternal blood oxygen/carbon dioxide, nutrients and wastes with fetal blood mainly takes place in the labyrinth zone [26]. Exposure of TCDD significantly constricted the labyrinth zone and thickened the basal zone, accompanying with the decrease in the diameter of maternal sinusoids and reduced maternal blood flow. Such altered vascular development in the placentas by TCDD-exposed dams may partially contribute to poor fetal growth and neonatal survival. Since ITE at the dose studies did not induce such alterations but activated CYP1A1 as TCDD did, we speculated that the signal downstream of the AhR/CYP1A1 might be different from the TCDD induced one [23].

Meanwhile, we did not observe significant difference in the density of fetal capillaries labeled by CD31 in the labyrinth zone among ITE, TCDD, and the vehicle-control. Given that ITE and TCDD differentially altered the maternal sinusoids, but not fetal capillaries in the development of the placental vasculature, we further examined the profile of placenta angiogenic factors and their receptors on GD20. The previous study demonstrated that the TCDD-exposed placentas were in an ischemic state at the late phase of pregnancy [19], which directly caused up-regulation of HIF-1α and activated the transcription of many genes involved in angiogenesis [30], [31]. In agreement with these observations, our current study exhibited that the expression of HIF-1α mRNA was elevated with the increasing dose of TCDD. Although high dose ITE also increased HIF-1α mRNA expression, it was probably due to the direct regulation of AhR ligands on the HIF-1α expression [32].

Regarding the VEGF/VEGFR system, VEGFs and their receptors VEGFRs play a critical role in the establishment and remodeling of placental blood vessels. The present results showed that administration of TCDD and ITE differentially elevated the transcription of VEGF-A, VEGF-B and their receptors. This different induction of such angiogenic factors might attribute to the induction of HIF-1α [33], [34]. Moreover, the expression of PIGF mRNA was only significantly up-regulated in both TCDD-exposed groups, which was consistent with the nonredundant roles of PIGF in tissue ischemia, malignancy and several disease conditions [35].

In addition, the expression of Ang/Tie2 system, which plays a pivotal role in vascular remodeling [7], [21], [36], was also described in the current study. It was proposed that Ang-1 serves as an agonist ligand, while Ang-2 serves as an antagonist ligand of their common receptor Tie2, which is expressed on endothelial cells [36]–[38]. The increased Ang-1 and Tie2 mRNA transcriptions were detected in both ITE- and TCDD-exposed placentas, whereas the expression of Ang-2 mRNA was enhanced only in TCDD-exposed placentas. Therefore, we proposed that the elevated expression of Ang-2 mRNA following exposure to TCDD was due to the ischemia induced by TCDD and might partially contribute to the morphological alterations in the labyrinth zone on GD20. This explanation was also demonstrated by the recent report that the expression of Ang-2 mRNA was induced under the ischemic conditions whereas the expression of Ang-1 and Tie2 mRNA remained unchanged in primary human endothelial cells and rat brain tissues [33], [39]. Most importantly, in the current study, two anti-angiogenic factors Endoglin and IFN-γ were notably elevated only in TCDD-exposed groups compared with both ITE-exposed groups and vehicle-control, which might be a consequence of ischemic conditions by TCDD administration [40] and interaction with other angiogenic-associated genes expression [41].

In conclusion, in the current study we compared putative endogenous AhR ligand ITE, and the prototypical xenobiotic ligand TCDD in the regulating the placental vascular remodeling. We found that in utero exposure to ITE or TCDD has distinct effects on fetal survival, differentially regulates placenta morphologic changes in the maternal side and the angiogenic-associated factors. Meanwhile, the dose or the exposure time of TCDD administration might not enough to induce the significant alteration in the density of fetal capillaries, placental weight and fetal weight. Thus, we speculated that the increase incidence of fetal death by exposure to TCDD is possibly due to the reduced blood flow and alterations of vascular morphologies in the placental labyrinth zone. Moreover, we also hypothesized that endogenous AhR ligand ITE and the toxic ligand TCDD may modify the transcription of different sets of genes to account for their different toxicity.
